# Supplementary figures and images for: Infrared and visible image fusion algorithm based on gradient attention residuals dense block
Source: PeerJ Comput Sci. 2024 Nov 28;10:e2569. doi: 10.7717/peerj-cs.2569 (PMC11622899; doi:10.7717/peerj-cs.2569)

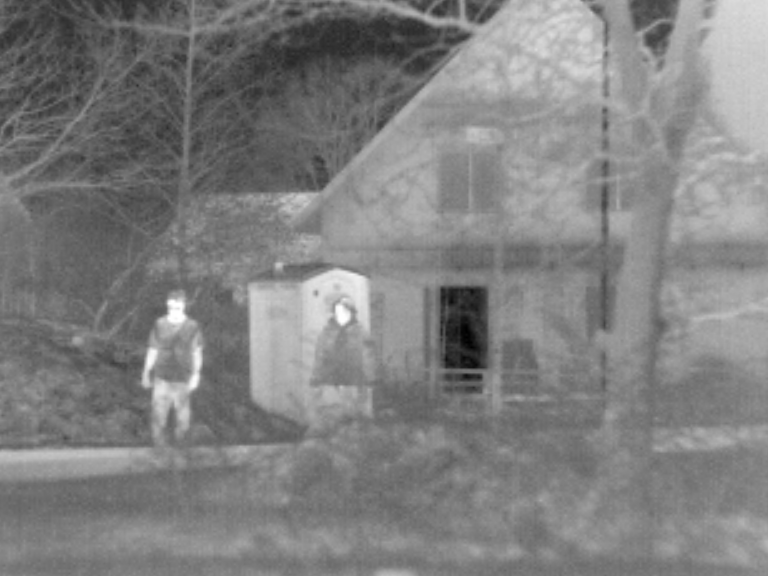

Supplement: Supplemental Information 3 — The data set is divided into infrared images and visible images, each type of image has 42, and one-to-one correspondence. [file peerj-cs-10-2569-s003.zip › TNO dataset/ir/01.png]

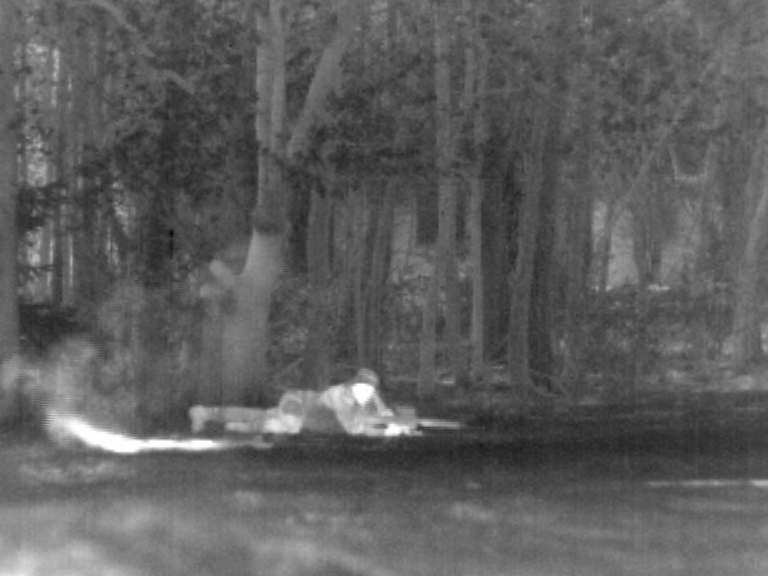

Supplement: Supplemental Information 3 — The data set is divided into infrared images and visible images, each type of image has 42, and one-to-one correspondence. [file peerj-cs-10-2569-s003.zip › TNO dataset/ir/02.png]

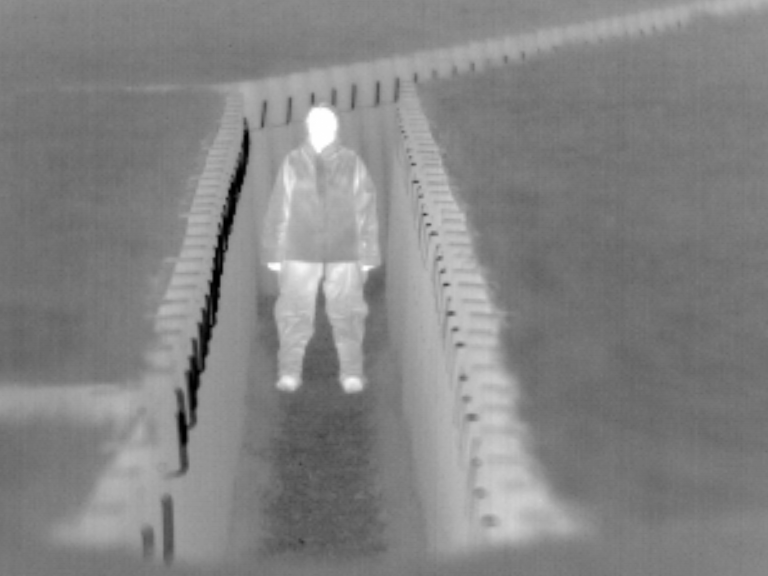

Supplement: Supplemental Information 3 — The data set is divided into infrared images and visible images, each type of image has 42, and one-to-one correspondence. [file peerj-cs-10-2569-s003.zip › TNO dataset/ir/03.png]

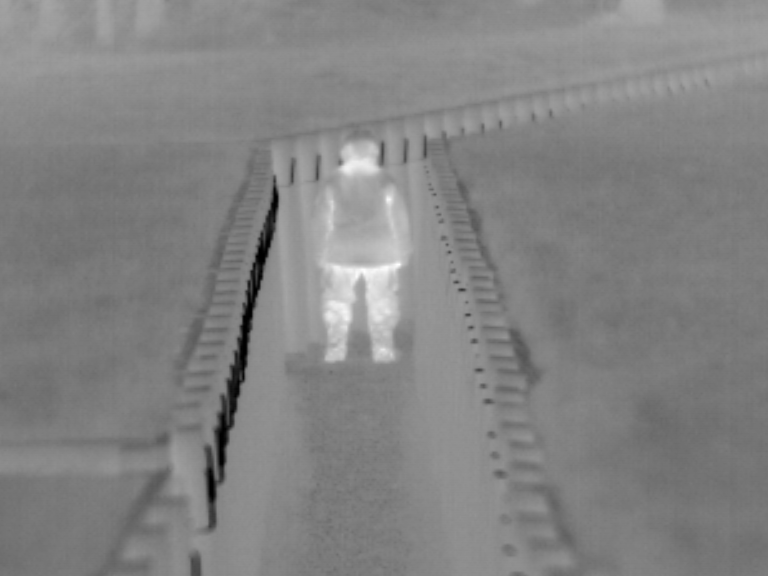

Supplement: Supplemental Information 3 — The data set is divided into infrared images and visible images, each type of image has 42, and one-to-one correspondence. [file peerj-cs-10-2569-s003.zip › TNO dataset/ir/04.png]

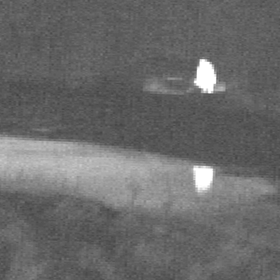

Supplement: Supplemental Information 3 — The data set is divided into infrared images and visible images, each type of image has 42, and one-to-one correspondence. [file peerj-cs-10-2569-s003.zip › TNO dataset/ir/05.png]

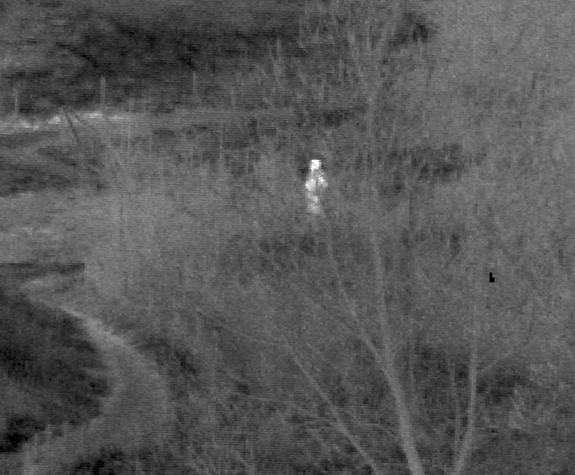

Supplement: Supplemental Information 3 — The data set is divided into infrared images and visible images, each type of image has 42, and one-to-one correspondence. [file peerj-cs-10-2569-s003.zip › TNO dataset/ir/06.png]

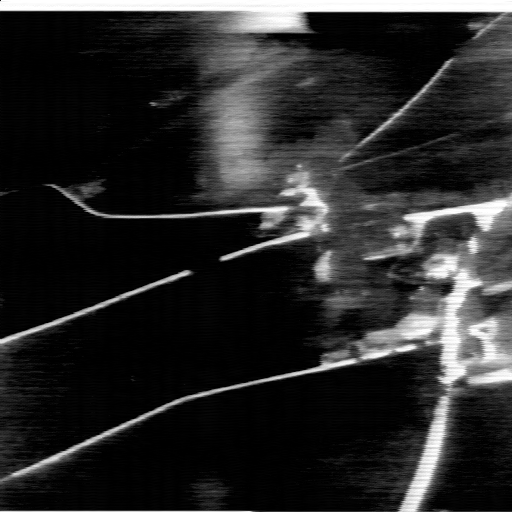

Supplement: Supplemental Information 3 — The data set is divided into infrared images and visible images, each type of image has 42, and one-to-one correspondence. [file peerj-cs-10-2569-s003.zip › TNO dataset/ir/07.png]

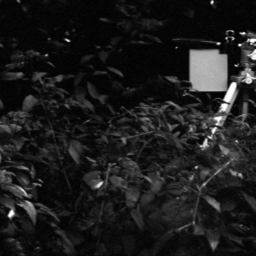

Supplement: Supplemental Information 3 — The data set is divided into infrared images and visible images, each type of image has 42, and one-to-one correspondence. [file peerj-cs-10-2569-s003.zip › TNO dataset/ir/08.png]

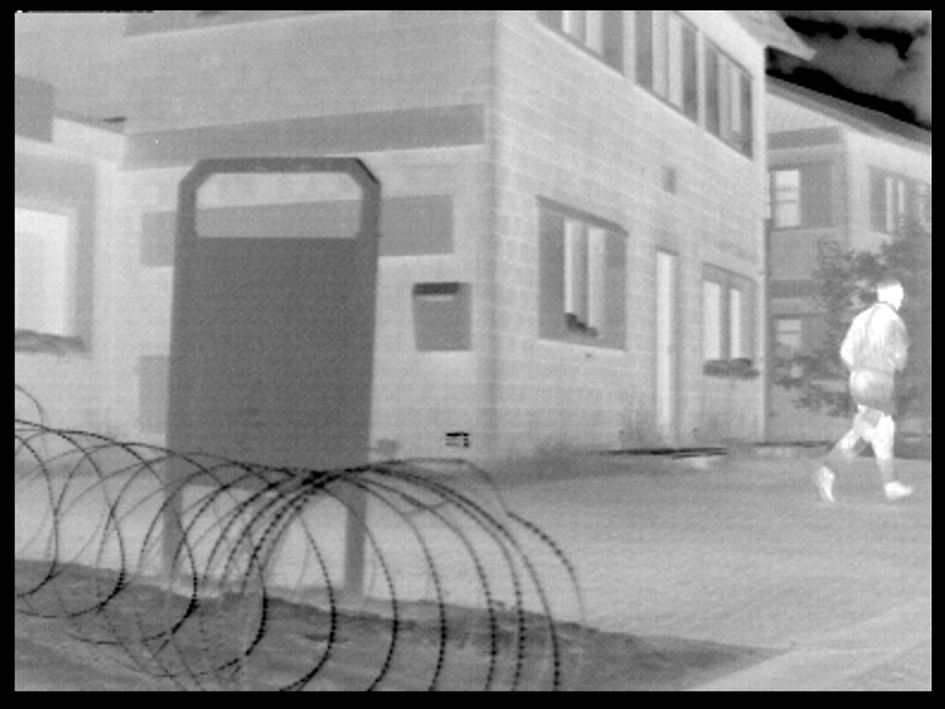

Supplement: Supplemental Information 3 — The data set is divided into infrared images and visible images, each type of image has 42, and one-to-one correspondence. [file peerj-cs-10-2569-s003.zip › TNO dataset/ir/09.png]

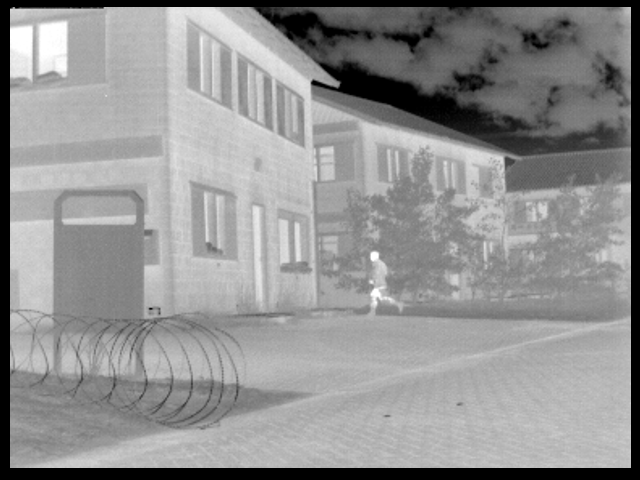

Supplement: Supplemental Information 3 — The data set is divided into infrared images and visible images, each type of image has 42, and one-to-one correspondence. [file peerj-cs-10-2569-s003.zip › TNO dataset/ir/10.png]

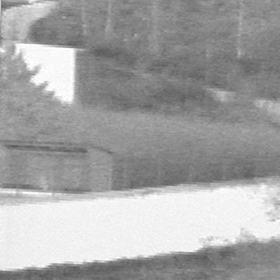

Supplement: Supplemental Information 3 — The data set is divided into infrared images and visible images, each type of image has 42, and one-to-one correspondence. [file peerj-cs-10-2569-s003.zip › TNO dataset/ir/11.png]

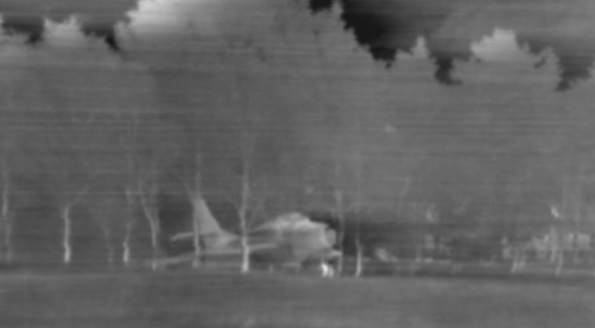

Supplement: Supplemental Information 3 — The data set is divided into infrared images and visible images, each type of image has 42, and one-to-one correspondence. [file peerj-cs-10-2569-s003.zip › TNO dataset/ir/12.png]

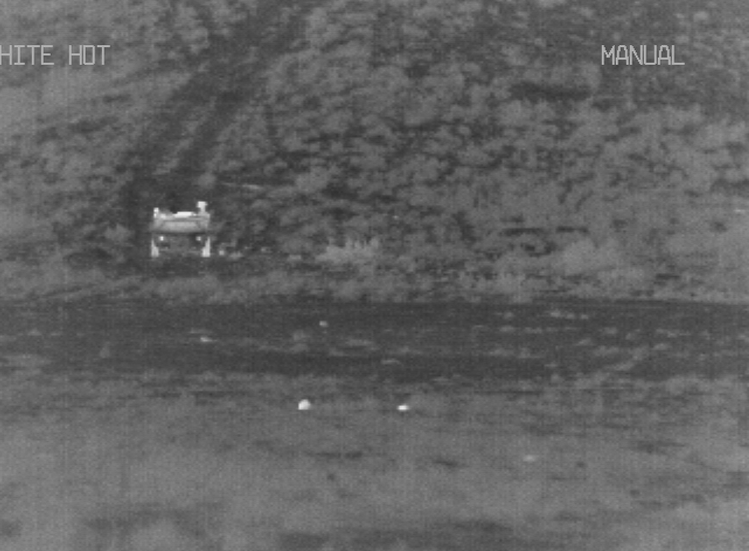

Supplement: Supplemental Information 3 — The data set is divided into infrared images and visible images, each type of image has 42, and one-to-one correspondence. [file peerj-cs-10-2569-s003.zip › TNO dataset/ir/13.png]

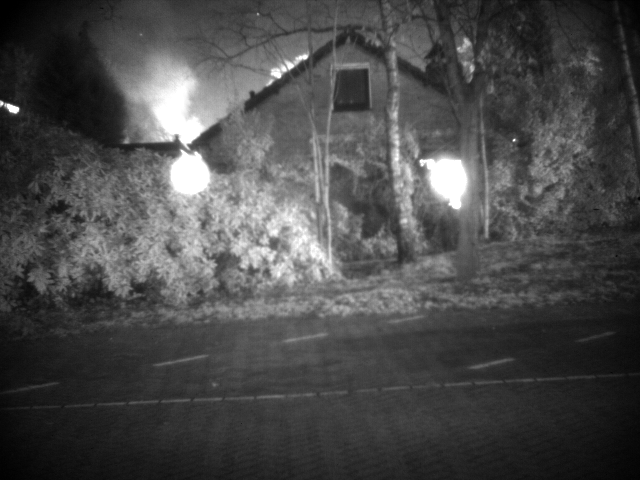

Supplement: Supplemental Information 3 — The data set is divided into infrared images and visible images, each type of image has 42, and one-to-one correspondence. [file peerj-cs-10-2569-s003.zip › TNO dataset/ir/14.png]

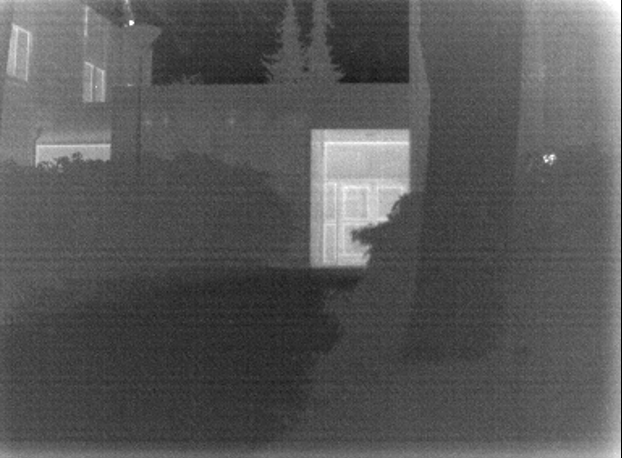

Supplement: Supplemental Information 3 — The data set is divided into infrared images and visible images, each type of image has 42, and one-to-one correspondence. [file peerj-cs-10-2569-s003.zip › TNO dataset/ir/15.png]

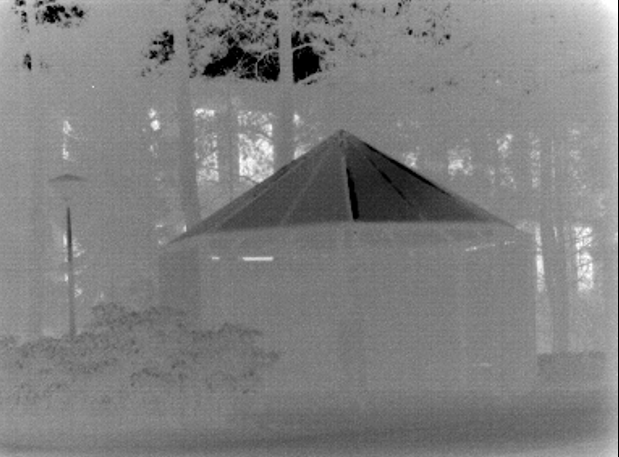

Supplement: Supplemental Information 3 — The data set is divided into infrared images and visible images, each type of image has 42, and one-to-one correspondence. [file peerj-cs-10-2569-s003.zip › TNO dataset/ir/16.png]

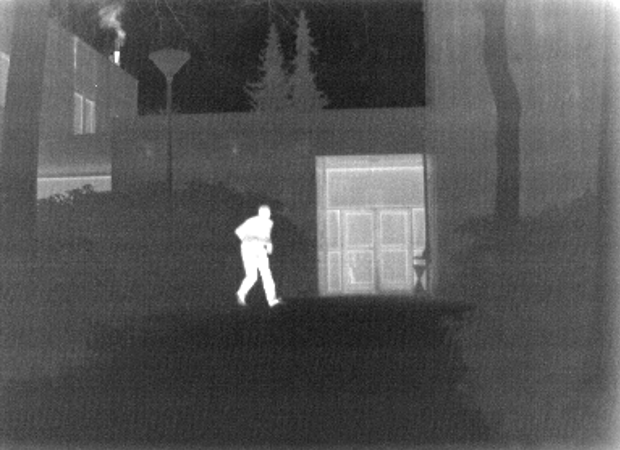

Supplement: Supplemental Information 3 — The data set is divided into infrared images and visible images, each type of image has 42, and one-to-one correspondence. [file peerj-cs-10-2569-s003.zip › TNO dataset/ir/17.png]

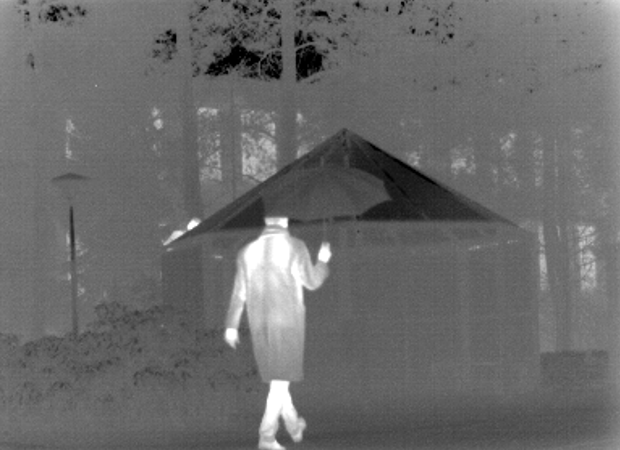

Supplement: Supplemental Information 3 — The data set is divided into infrared images and visible images, each type of image has 42, and one-to-one correspondence. [file peerj-cs-10-2569-s003.zip › TNO dataset/ir/18.png]

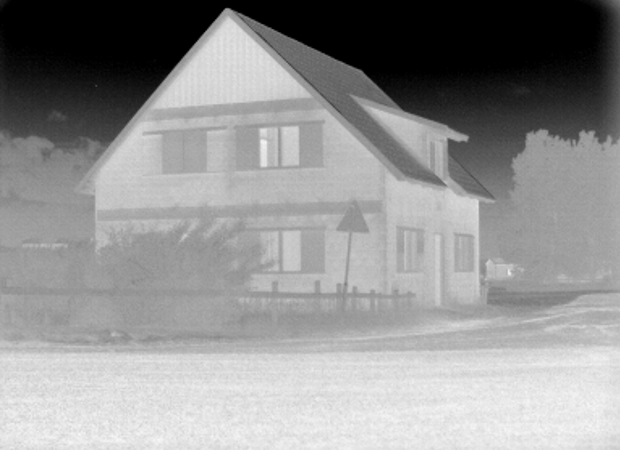

Supplement: Supplemental Information 3 — The data set is divided into infrared images and visible images, each type of image has 42, and one-to-one correspondence. [file peerj-cs-10-2569-s003.zip › TNO dataset/ir/19.png]

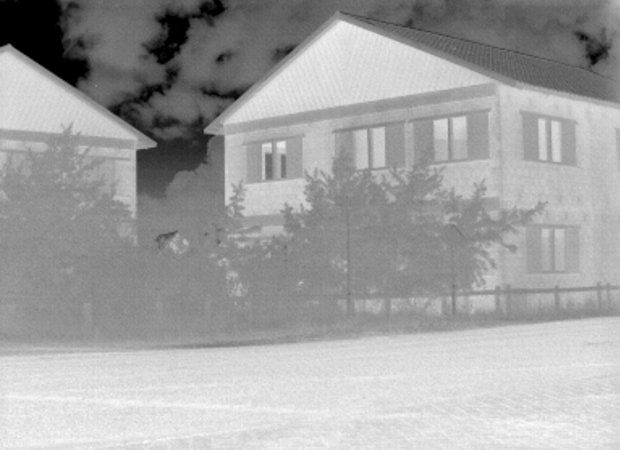

Supplement: Supplemental Information 3 — The data set is divided into infrared images and visible images, each type of image has 42, and one-to-one correspondence. [file peerj-cs-10-2569-s003.zip › TNO dataset/ir/20.png]

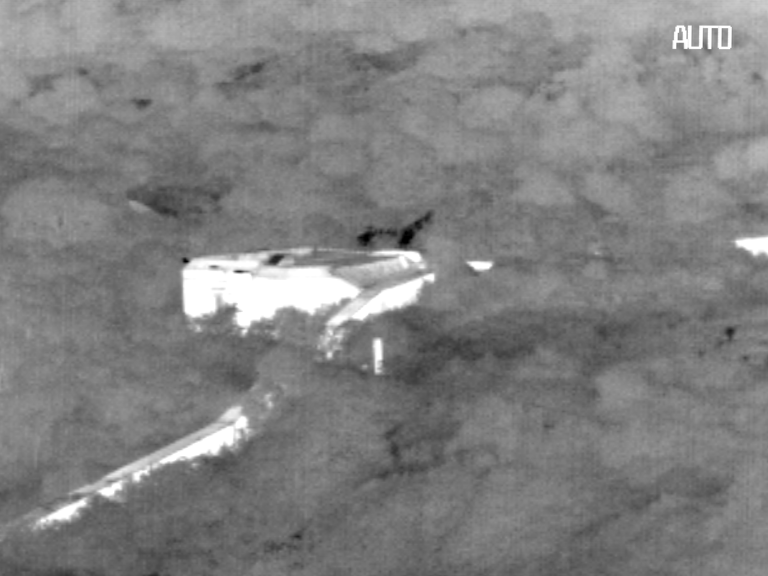

Supplement: Supplemental Information 3 — The data set is divided into infrared images and visible images, each type of image has 42, and one-to-one correspondence. [file peerj-cs-10-2569-s003.zip › TNO dataset/ir/21.png]

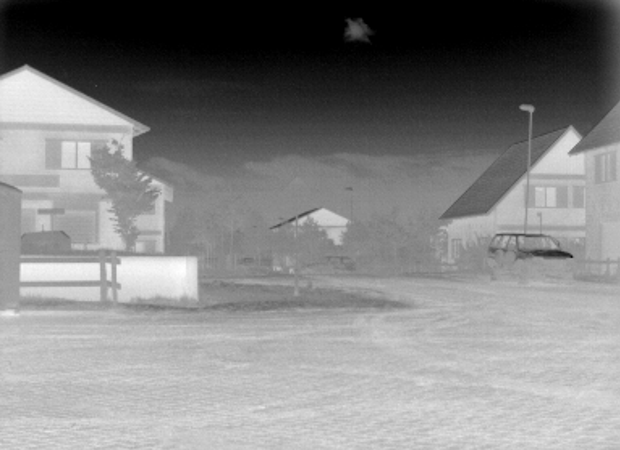

Supplement: Supplemental Information 3 — The data set is divided into infrared images and visible images, each type of image has 42, and one-to-one correspondence. [file peerj-cs-10-2569-s003.zip › TNO dataset/ir/22.png]

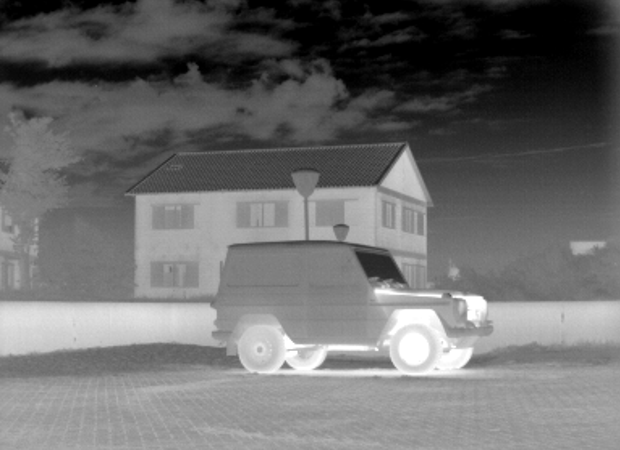

Supplement: Supplemental Information 3 — The data set is divided into infrared images and visible images, each type of image has 42, and one-to-one correspondence. [file peerj-cs-10-2569-s003.zip › TNO dataset/ir/23.png]

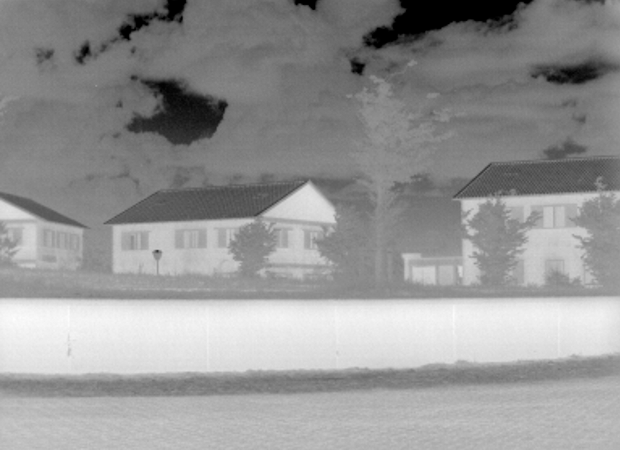

Supplement: Supplemental Information 3 — The data set is divided into infrared images and visible images, each type of image has 42, and one-to-one correspondence. [file peerj-cs-10-2569-s003.zip › TNO dataset/ir/24.png]

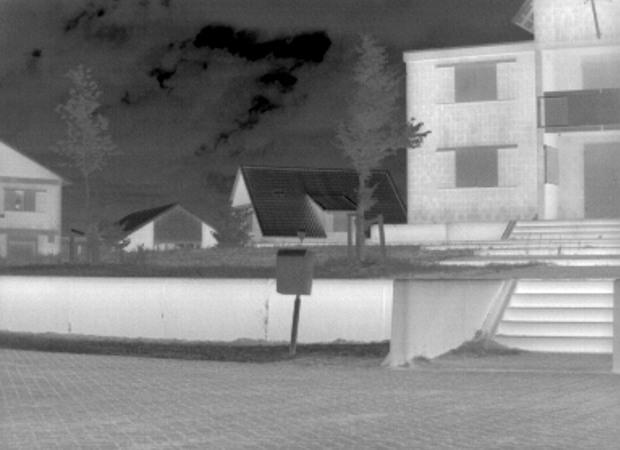

Supplement: Supplemental Information 3 — The data set is divided into infrared images and visible images, each type of image has 42, and one-to-one correspondence. [file peerj-cs-10-2569-s003.zip › TNO dataset/ir/25.png]

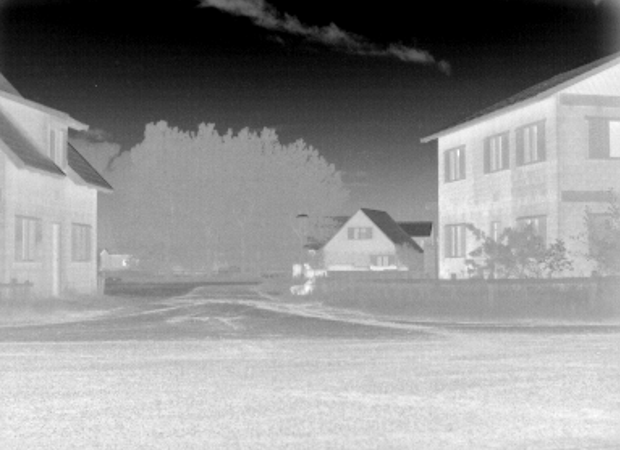

Supplement: Supplemental Information 3 — The data set is divided into infrared images and visible images, each type of image has 42, and one-to-one correspondence. [file peerj-cs-10-2569-s003.zip › TNO dataset/ir/26.png]

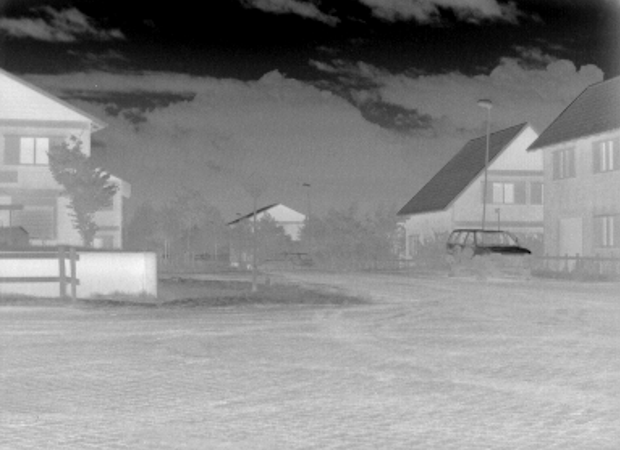

Supplement: Supplemental Information 3 — The data set is divided into infrared images and visible images, each type of image has 42, and one-to-one correspondence. [file peerj-cs-10-2569-s003.zip › TNO dataset/ir/27.png]

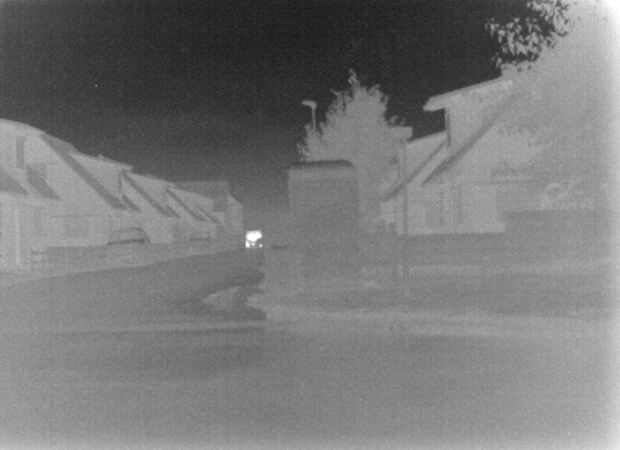

Supplement: Supplemental Information 3 — The data set is divided into infrared images and visible images, each type of image has 42, and one-to-one correspondence. [file peerj-cs-10-2569-s003.zip › TNO dataset/ir/28.png]

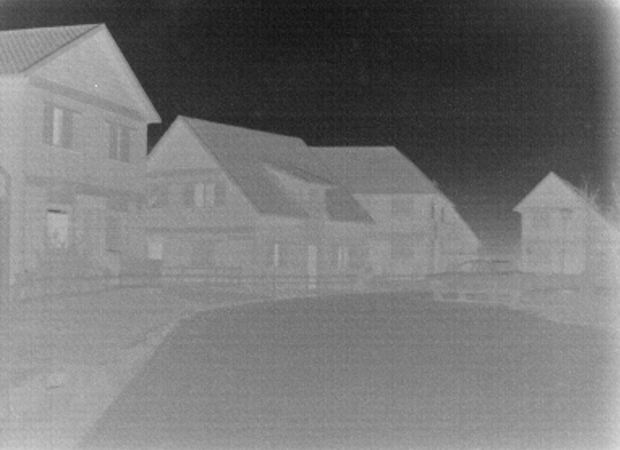

Supplement: Supplemental Information 3 — The data set is divided into infrared images and visible images, each type of image has 42, and one-to-one correspondence. [file peerj-cs-10-2569-s003.zip › TNO dataset/ir/29.png]

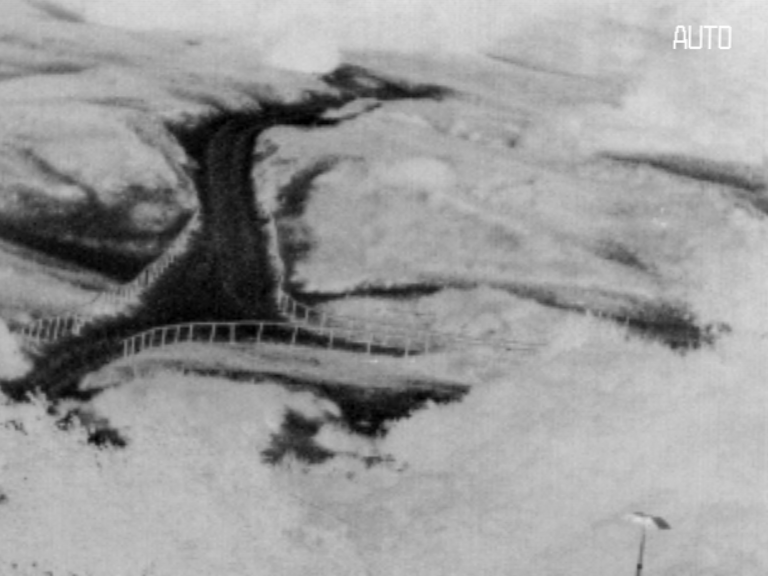

Supplement: Supplemental Information 3 — The data set is divided into infrared images and visible images, each type of image has 42, and one-to-one correspondence. [file peerj-cs-10-2569-s003.zip › TNO dataset/ir/30.png]

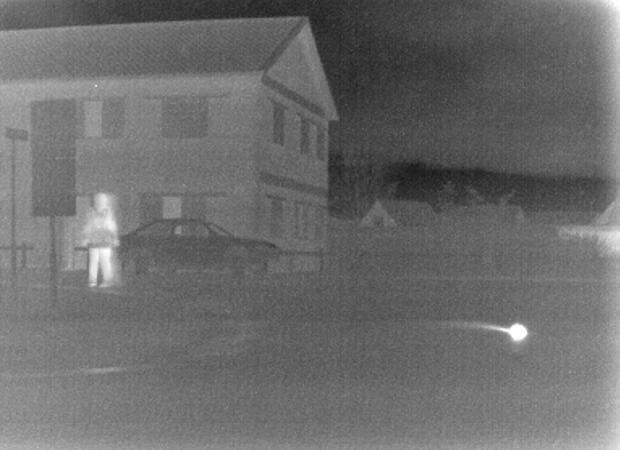

Supplement: Supplemental Information 3 — The data set is divided into infrared images and visible images, each type of image has 42, and one-to-one correspondence. [file peerj-cs-10-2569-s003.zip › TNO dataset/ir/31.png]

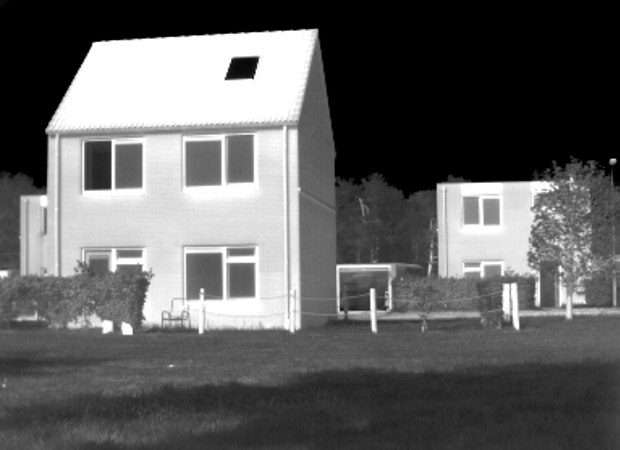

Supplement: Supplemental Information 3 — The data set is divided into infrared images and visible images, each type of image has 42, and one-to-one correspondence. [file peerj-cs-10-2569-s003.zip › TNO dataset/ir/32.png]

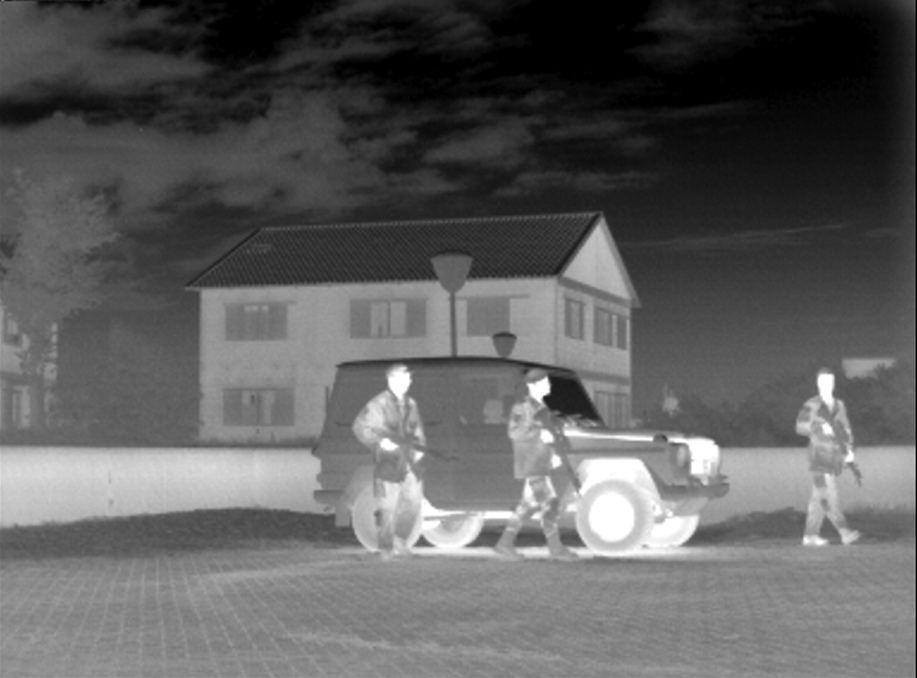

Supplement: Supplemental Information 3 — The data set is divided into infrared images and visible images, each type of image has 42, and one-to-one correspondence. [file peerj-cs-10-2569-s003.zip › TNO dataset/ir/33.png]

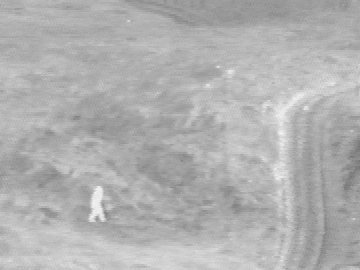

Supplement: Supplemental Information 3 — The data set is divided into infrared images and visible images, each type of image has 42, and one-to-one correspondence. [file peerj-cs-10-2569-s003.zip › TNO dataset/ir/34.png]

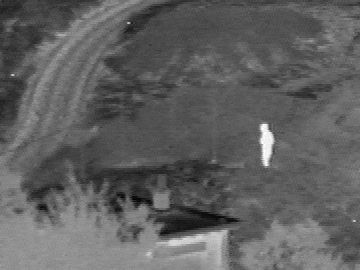

Supplement: Supplemental Information 3 — The data set is divided into infrared images and visible images, each type of image has 42, and one-to-one correspondence. [file peerj-cs-10-2569-s003.zip › TNO dataset/ir/35.png]

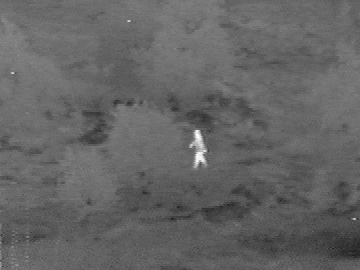

Supplement: Supplemental Information 3 — The data set is divided into infrared images and visible images, each type of image has 42, and one-to-one correspondence. [file peerj-cs-10-2569-s003.zip › TNO dataset/ir/36.png]

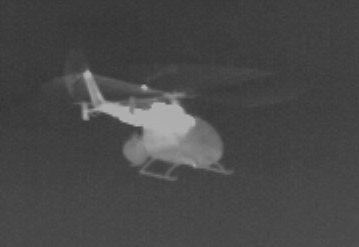

Supplement: Supplemental Information 3 — The data set is divided into infrared images and visible images, each type of image has 42, and one-to-one correspondence. [file peerj-cs-10-2569-s003.zip › TNO dataset/ir/37.png]

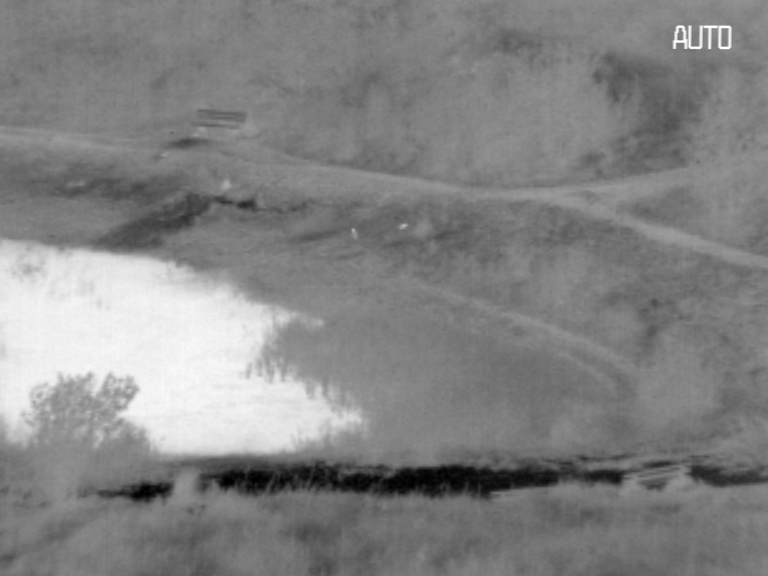

Supplement: Supplemental Information 3 — The data set is divided into infrared images and visible images, each type of image has 42, and one-to-one correspondence. [file peerj-cs-10-2569-s003.zip › TNO dataset/ir/38.png]

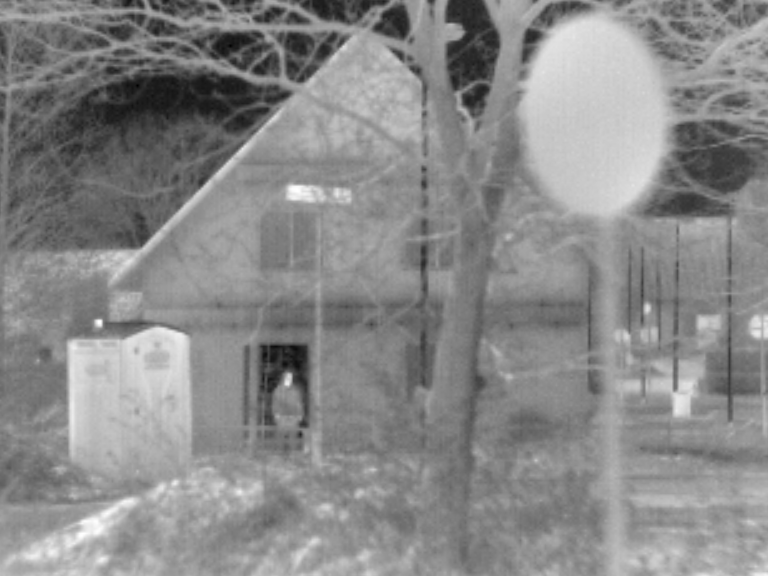

Supplement: Supplemental Information 3 — The data set is divided into infrared images and visible images, each type of image has 42, and one-to-one correspondence. [file peerj-cs-10-2569-s003.zip › TNO dataset/ir/39.png]

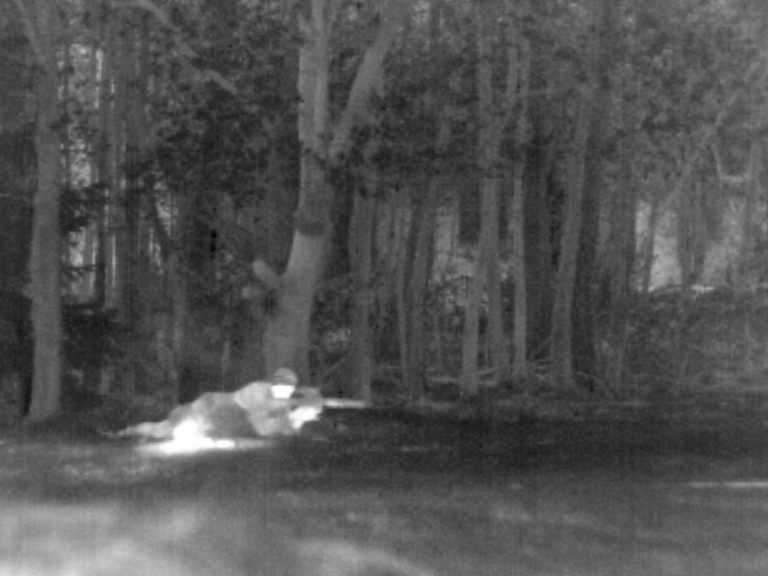

Supplement: Supplemental Information 3 — The data set is divided into infrared images and visible images, each type of image has 42, and one-to-one correspondence. [file peerj-cs-10-2569-s003.zip › TNO dataset/ir/40.png]

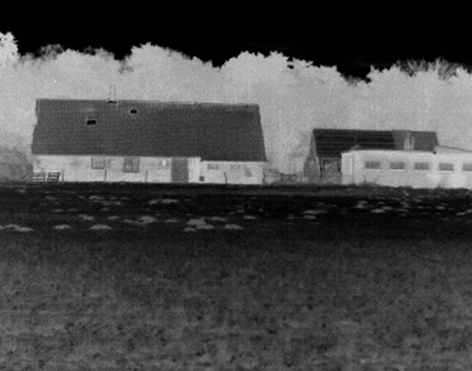

Supplement: Supplemental Information 3 — The data set is divided into infrared images and visible images, each type of image has 42, and one-to-one correspondence. [file peerj-cs-10-2569-s003.zip › TNO dataset/ir/41.png]

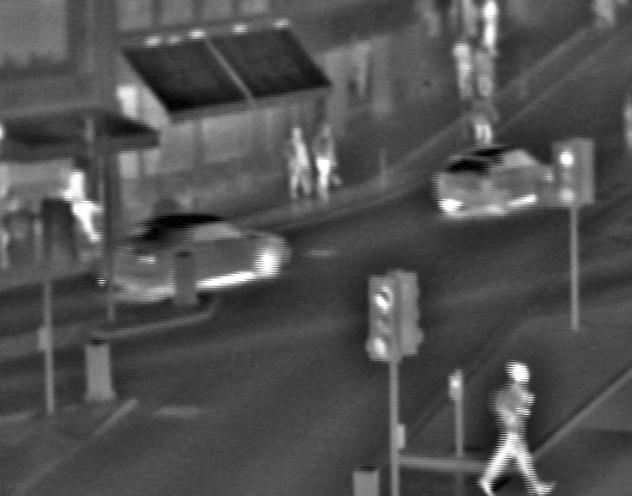

Supplement: Supplemental Information 3 — The data set is divided into infrared images and visible images, each type of image has 42, and one-to-one correspondence. [file peerj-cs-10-2569-s003.zip › TNO dataset/ir/42.png]

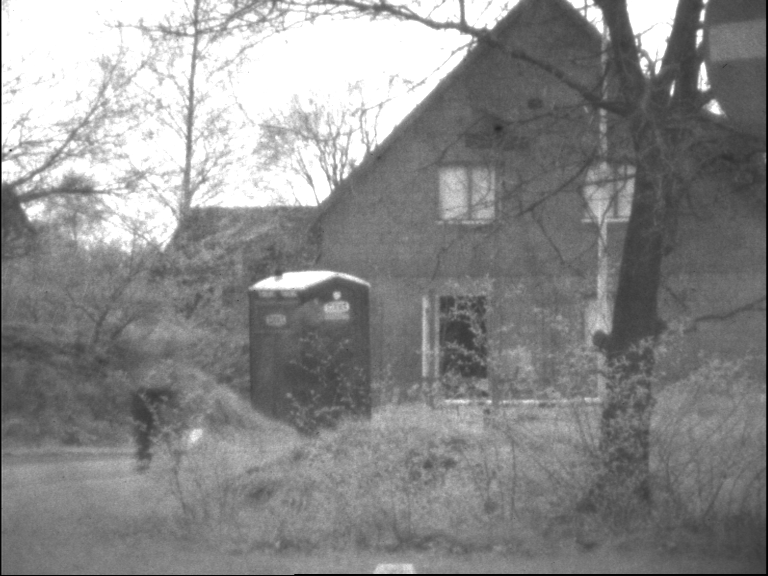

Supplement: Supplemental Information 3 — The data set is divided into infrared images and visible images, each type of image has 42, and one-to-one correspondence. [file peerj-cs-10-2569-s003.zip › TNO dataset/vi/01.png]

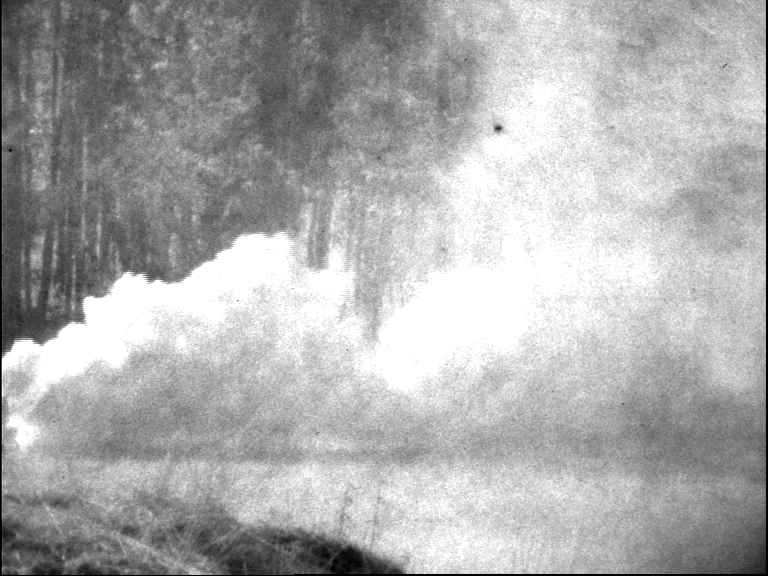

Supplement: Supplemental Information 3 — The data set is divided into infrared images and visible images, each type of image has 42, and one-to-one correspondence. [file peerj-cs-10-2569-s003.zip › TNO dataset/vi/02.png]

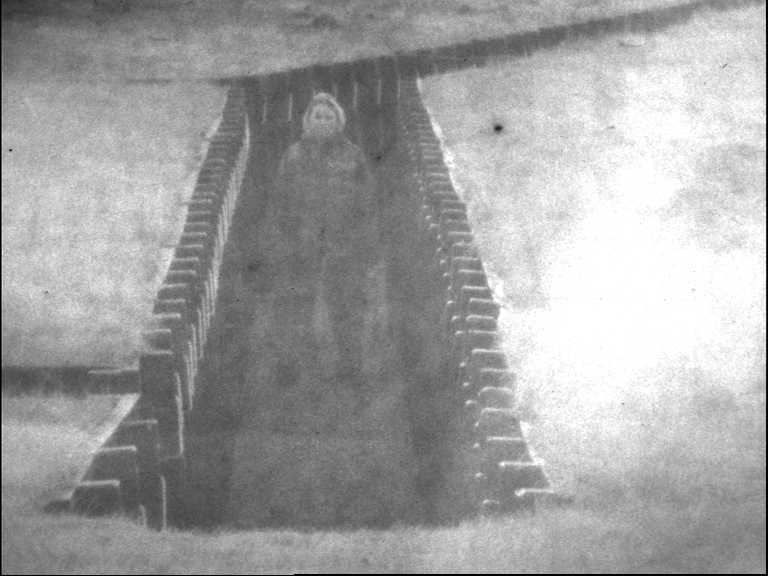

Supplement: Supplemental Information 3 — The data set is divided into infrared images and visible images, each type of image has 42, and one-to-one correspondence. [file peerj-cs-10-2569-s003.zip › TNO dataset/vi/03.png]

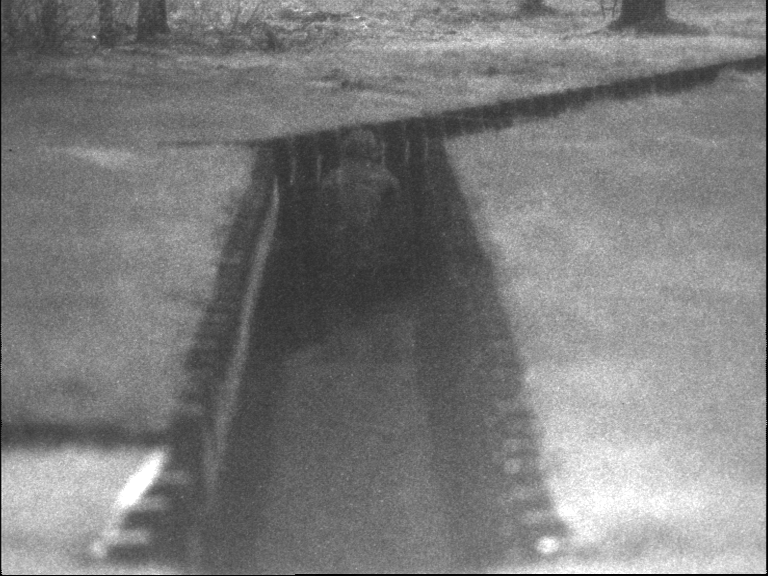

Supplement: Supplemental Information 3 — The data set is divided into infrared images and visible images, each type of image has 42, and one-to-one correspondence. [file peerj-cs-10-2569-s003.zip › TNO dataset/vi/04.png]

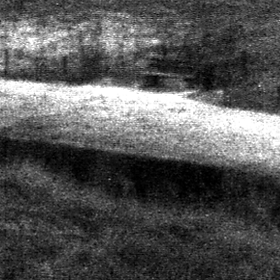

Supplement: Supplemental Information 3 — The data set is divided into infrared images and visible images, each type of image has 42, and one-to-one correspondence. [file peerj-cs-10-2569-s003.zip › TNO dataset/vi/05.png]

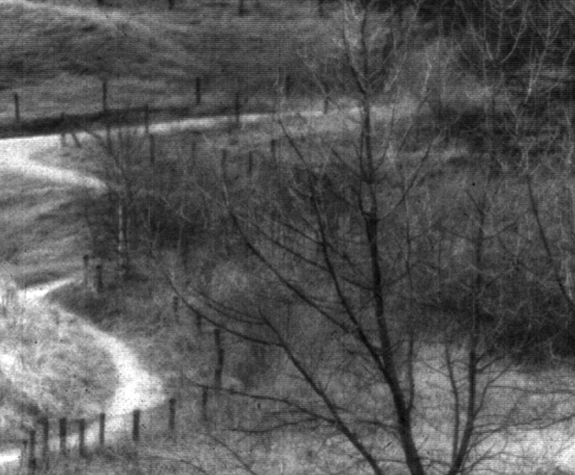

Supplement: Supplemental Information 3 — The data set is divided into infrared images and visible images, each type of image has 42, and one-to-one correspondence. [file peerj-cs-10-2569-s003.zip › TNO dataset/vi/06.png]

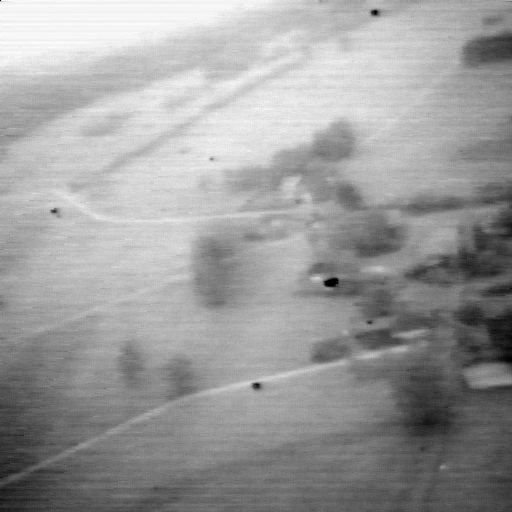

Supplement: Supplemental Information 3 — The data set is divided into infrared images and visible images, each type of image has 42, and one-to-one correspondence. [file peerj-cs-10-2569-s003.zip › TNO dataset/vi/07.png]

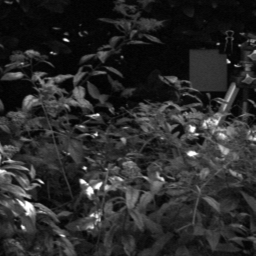

Supplement: Supplemental Information 3 — The data set is divided into infrared images and visible images, each type of image has 42, and one-to-one correspondence. [file peerj-cs-10-2569-s003.zip › TNO dataset/vi/08.png]

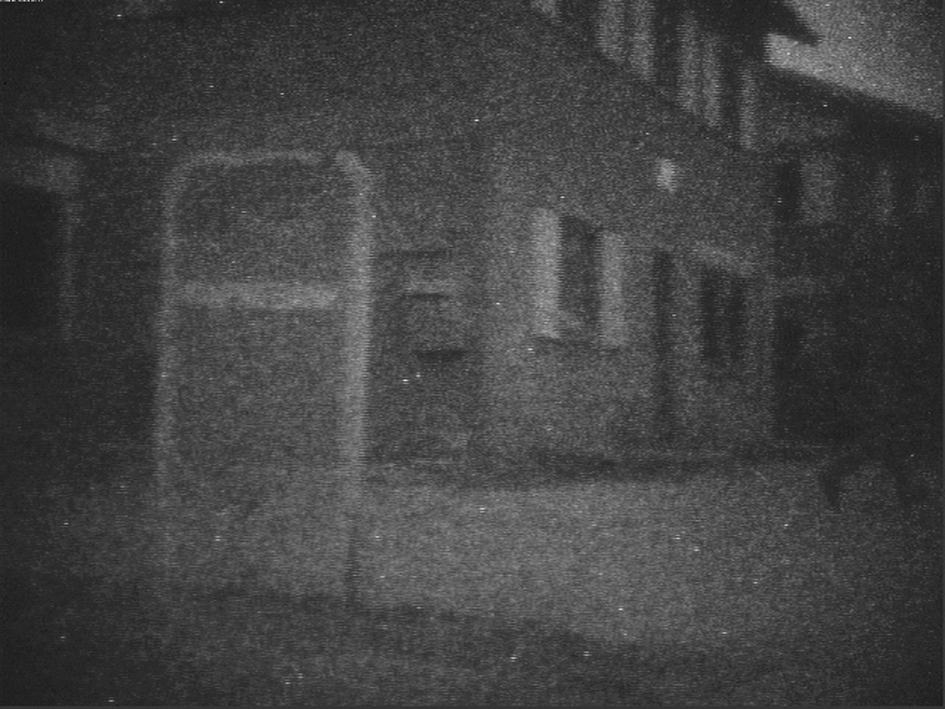

Supplement: Supplemental Information 3 — The data set is divided into infrared images and visible images, each type of image has 42, and one-to-one correspondence. [file peerj-cs-10-2569-s003.zip › TNO dataset/vi/09.png]

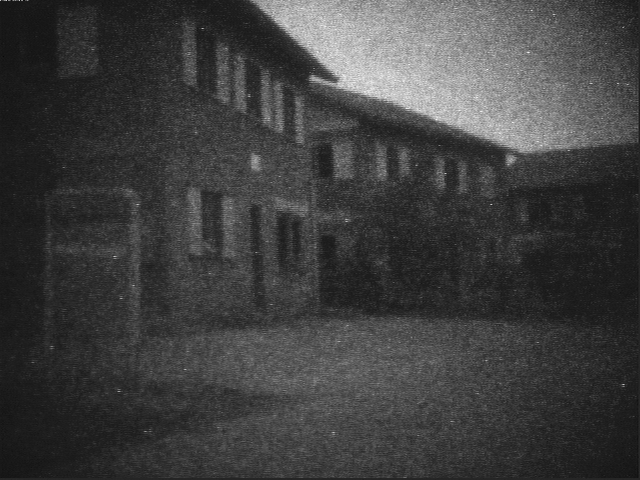

Supplement: Supplemental Information 3 — The data set is divided into infrared images and visible images, each type of image has 42, and one-to-one correspondence. [file peerj-cs-10-2569-s003.zip › TNO dataset/vi/10.png]

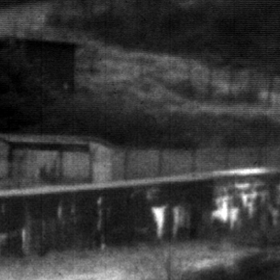

Supplement: Supplemental Information 3 — The data set is divided into infrared images and visible images, each type of image has 42, and one-to-one correspondence. [file peerj-cs-10-2569-s003.zip › TNO dataset/vi/11.png]

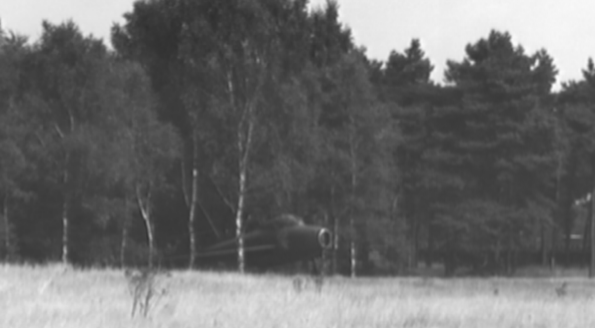

Supplement: Supplemental Information 3 — The data set is divided into infrared images and visible images, each type of image has 42, and one-to-one correspondence. [file peerj-cs-10-2569-s003.zip › TNO dataset/vi/12.png]

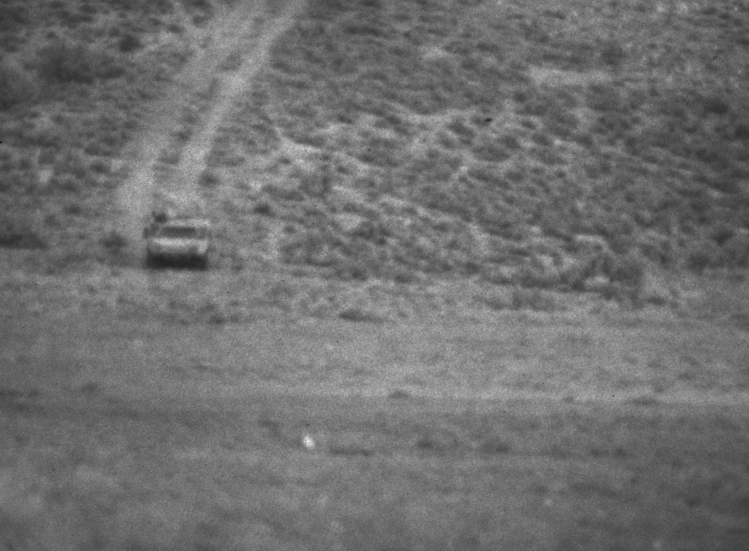

Supplement: Supplemental Information 3 — The data set is divided into infrared images and visible images, each type of image has 42, and one-to-one correspondence. [file peerj-cs-10-2569-s003.zip › TNO dataset/vi/13.png]

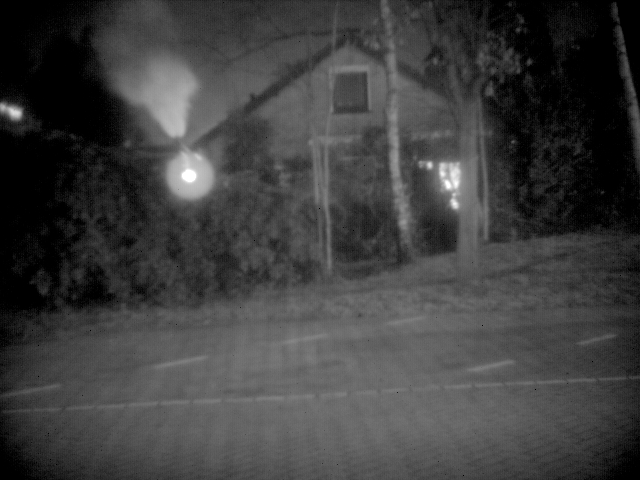

Supplement: Supplemental Information 3 — The data set is divided into infrared images and visible images, each type of image has 42, and one-to-one correspondence. [file peerj-cs-10-2569-s003.zip › TNO dataset/vi/14.png]

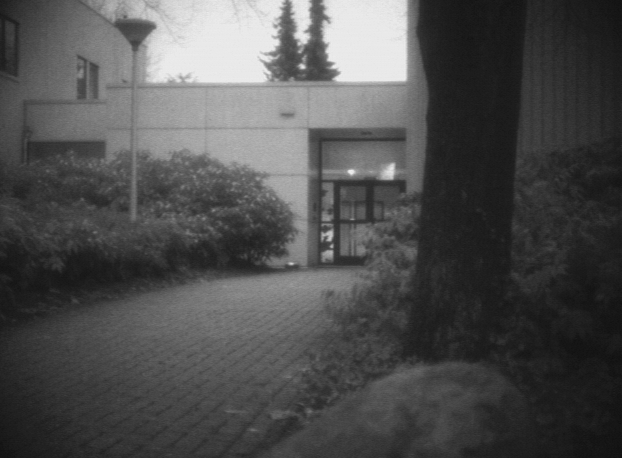

Supplement: Supplemental Information 3 — The data set is divided into infrared images and visible images, each type of image has 42, and one-to-one correspondence. [file peerj-cs-10-2569-s003.zip › TNO dataset/vi/15.png]

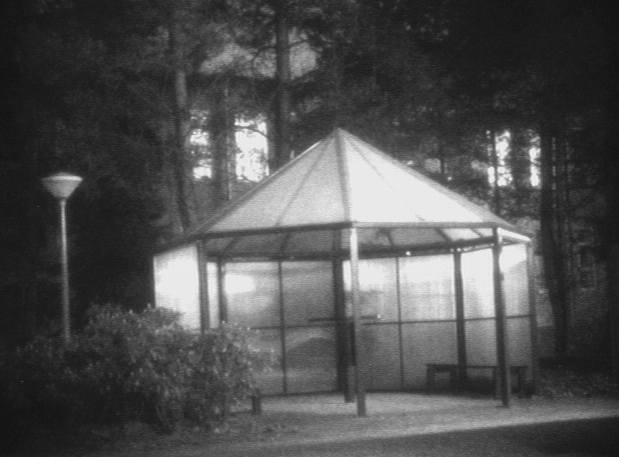

Supplement: Supplemental Information 3 — The data set is divided into infrared images and visible images, each type of image has 42, and one-to-one correspondence. [file peerj-cs-10-2569-s003.zip › TNO dataset/vi/16.png]

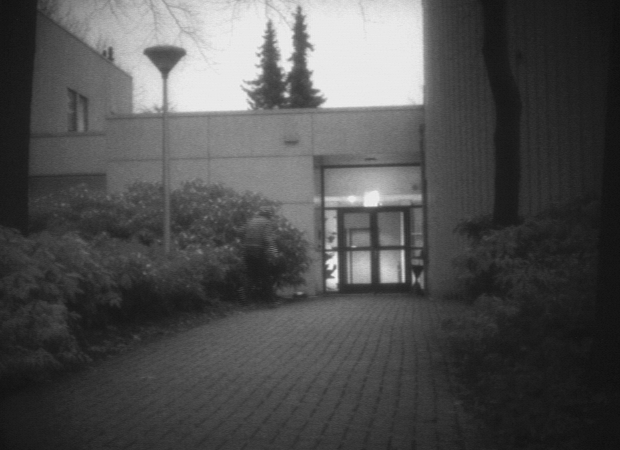

Supplement: Supplemental Information 3 — The data set is divided into infrared images and visible images, each type of image has 42, and one-to-one correspondence. [file peerj-cs-10-2569-s003.zip › TNO dataset/vi/17.png]

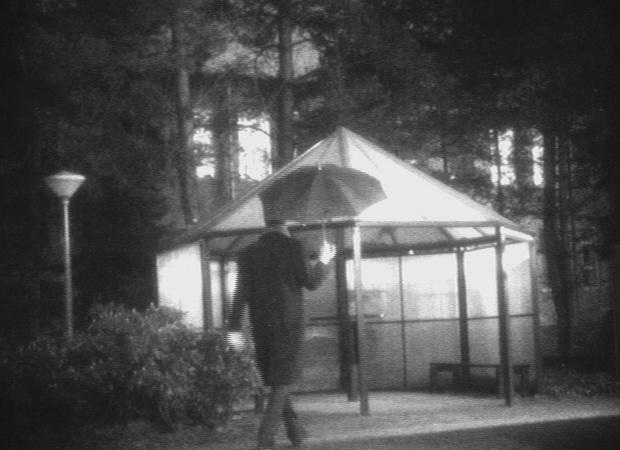

Supplement: Supplemental Information 3 — The data set is divided into infrared images and visible images, each type of image has 42, and one-to-one correspondence. [file peerj-cs-10-2569-s003.zip › TNO dataset/vi/18.png]

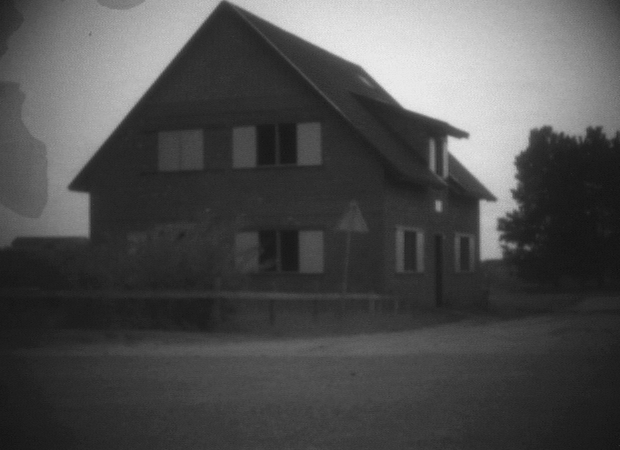

Supplement: Supplemental Information 3 — The data set is divided into infrared images and visible images, each type of image has 42, and one-to-one correspondence. [file peerj-cs-10-2569-s003.zip › TNO dataset/vi/19.png]

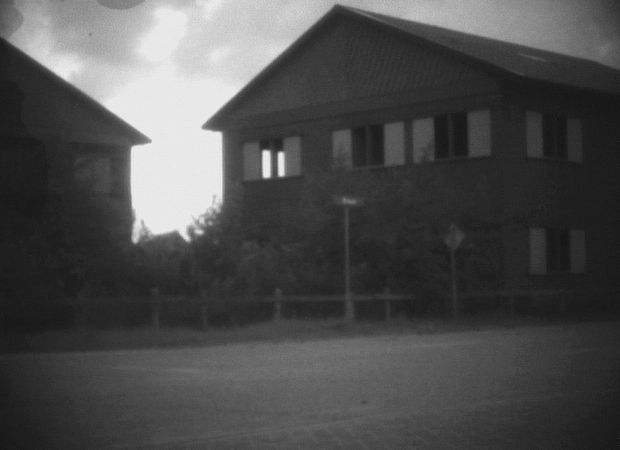

Supplement: Supplemental Information 3 — The data set is divided into infrared images and visible images, each type of image has 42, and one-to-one correspondence. [file peerj-cs-10-2569-s003.zip › TNO dataset/vi/20.png]

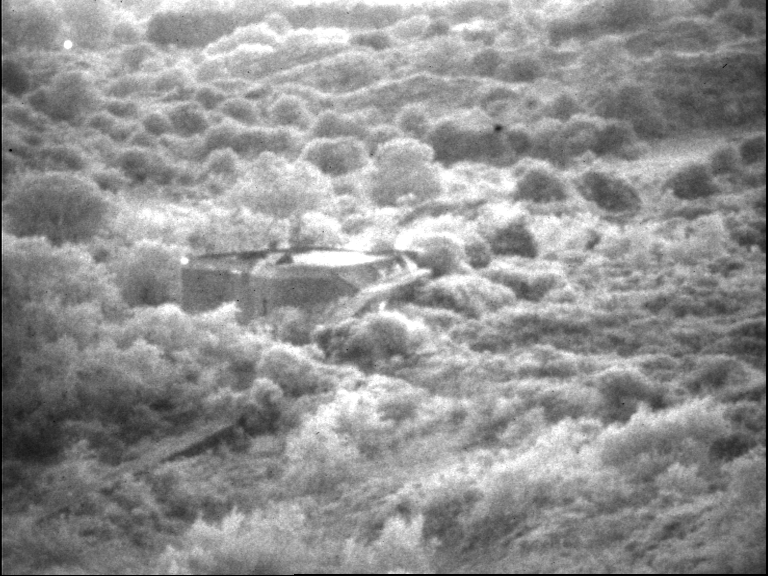

Supplement: Supplemental Information 3 — The data set is divided into infrared images and visible images, each type of image has 42, and one-to-one correspondence. [file peerj-cs-10-2569-s003.zip › TNO dataset/vi/21.png]

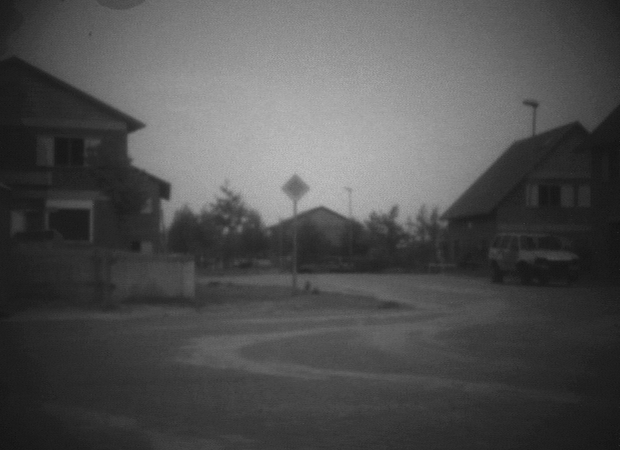

Supplement: Supplemental Information 3 — The data set is divided into infrared images and visible images, each type of image has 42, and one-to-one correspondence. [file peerj-cs-10-2569-s003.zip › TNO dataset/vi/22.png]

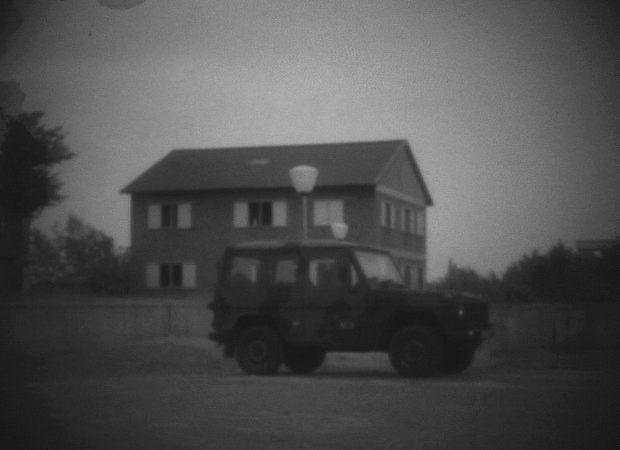

Supplement: Supplemental Information 3 — The data set is divided into infrared images and visible images, each type of image has 42, and one-to-one correspondence. [file peerj-cs-10-2569-s003.zip › TNO dataset/vi/23.png]

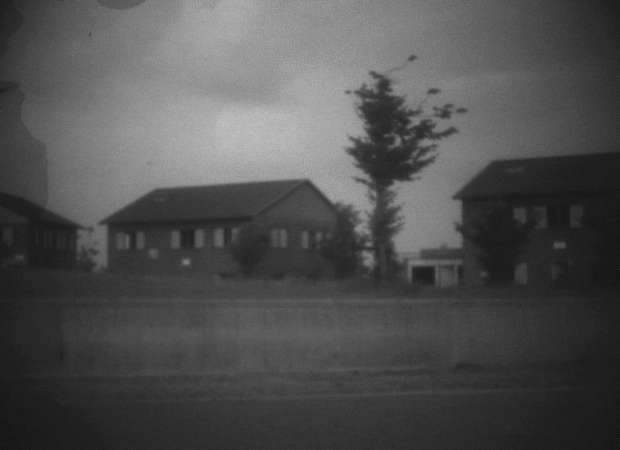

Supplement: Supplemental Information 3 — The data set is divided into infrared images and visible images, each type of image has 42, and one-to-one correspondence. [file peerj-cs-10-2569-s003.zip › TNO dataset/vi/24.png]

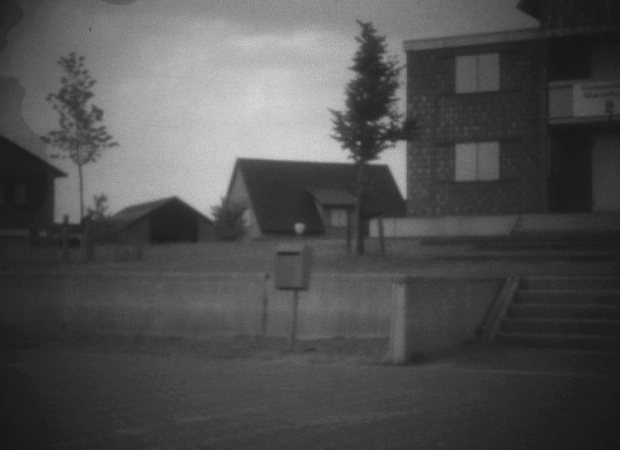

Supplement: Supplemental Information 3 — The data set is divided into infrared images and visible images, each type of image has 42, and one-to-one correspondence. [file peerj-cs-10-2569-s003.zip › TNO dataset/vi/25.png]

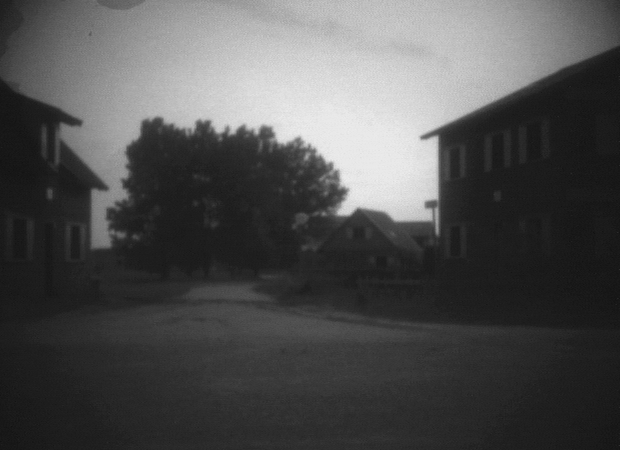

Supplement: Supplemental Information 3 — The data set is divided into infrared images and visible images, each type of image has 42, and one-to-one correspondence. [file peerj-cs-10-2569-s003.zip › TNO dataset/vi/26.png]

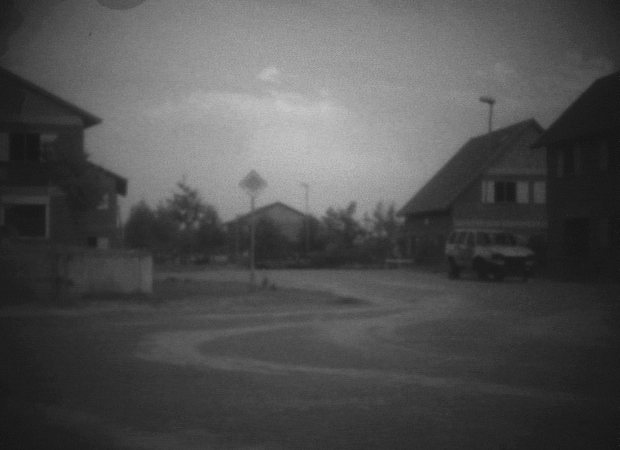

Supplement: Supplemental Information 3 — The data set is divided into infrared images and visible images, each type of image has 42, and one-to-one correspondence. [file peerj-cs-10-2569-s003.zip › TNO dataset/vi/27.png]

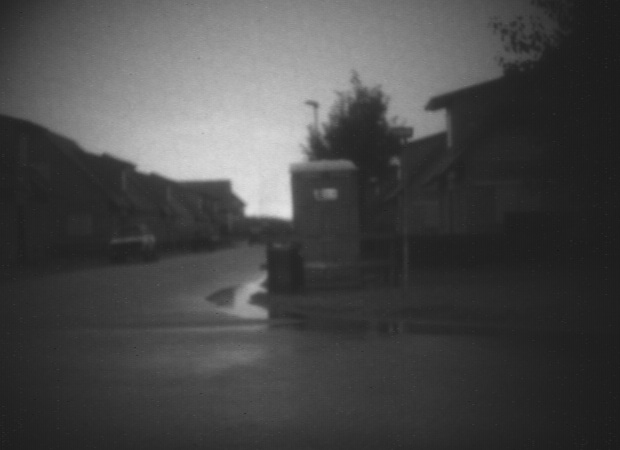

Supplement: Supplemental Information 3 — The data set is divided into infrared images and visible images, each type of image has 42, and one-to-one correspondence. [file peerj-cs-10-2569-s003.zip › TNO dataset/vi/28.png]

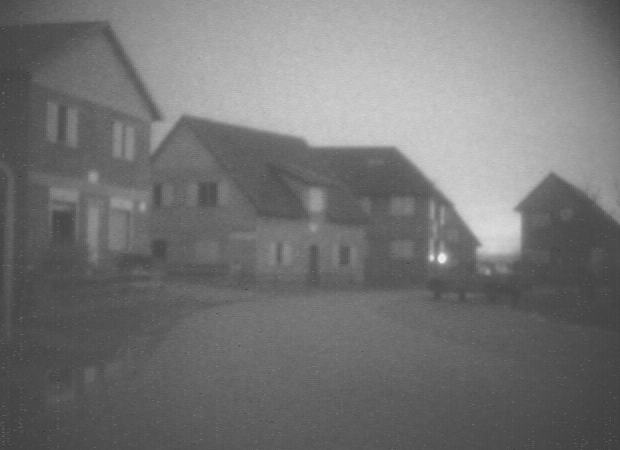

Supplement: Supplemental Information 3 — The data set is divided into infrared images and visible images, each type of image has 42, and one-to-one correspondence. [file peerj-cs-10-2569-s003.zip › TNO dataset/vi/29.png]

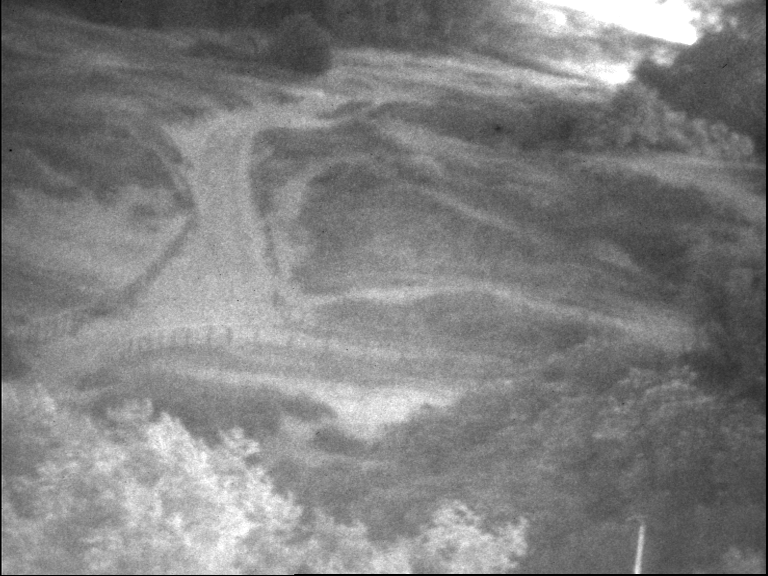

Supplement: Supplemental Information 3 — The data set is divided into infrared images and visible images, each type of image has 42, and one-to-one correspondence. [file peerj-cs-10-2569-s003.zip › TNO dataset/vi/30.png]

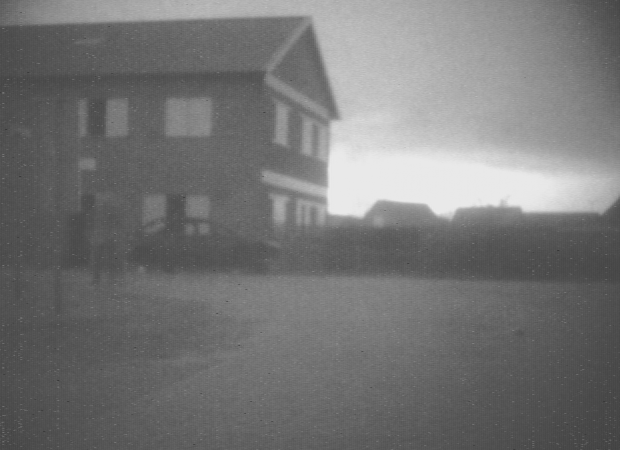

Supplement: Supplemental Information 3 — The data set is divided into infrared images and visible images, each type of image has 42, and one-to-one correspondence. [file peerj-cs-10-2569-s003.zip › TNO dataset/vi/31.png]

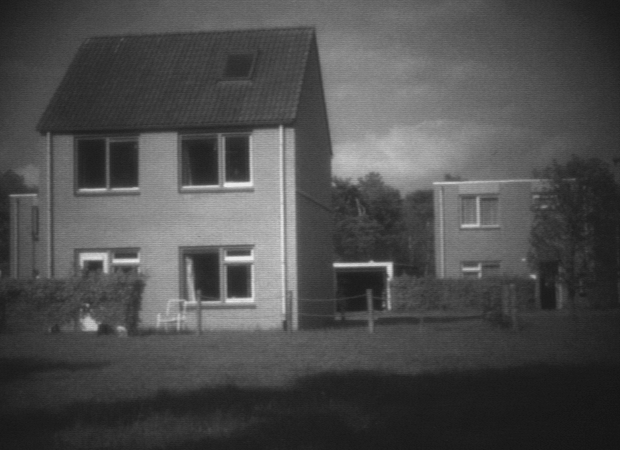

Supplement: Supplemental Information 3 — The data set is divided into infrared images and visible images, each type of image has 42, and one-to-one correspondence. [file peerj-cs-10-2569-s003.zip › TNO dataset/vi/32.png]

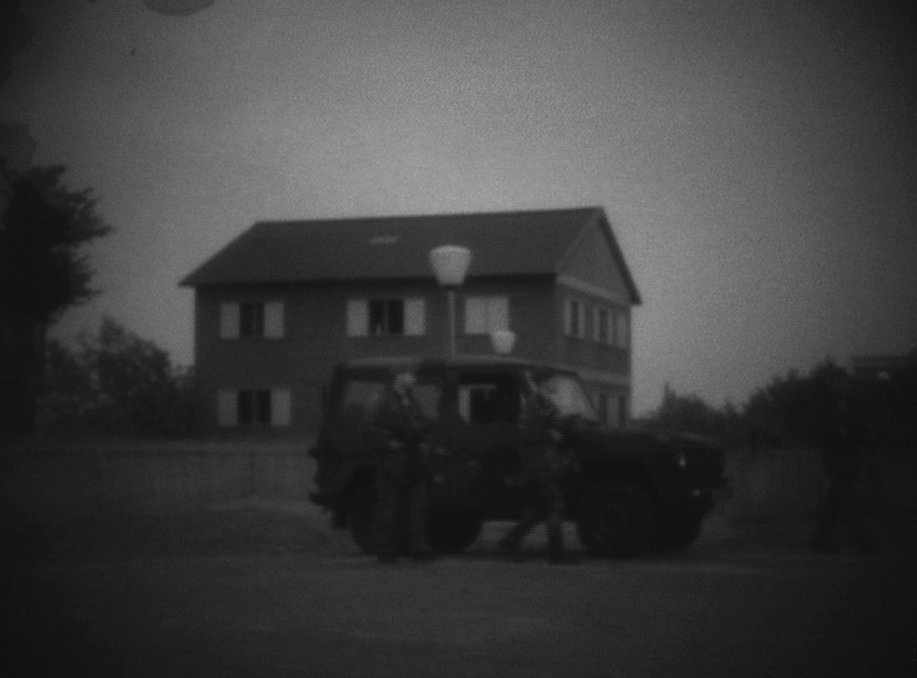

Supplement: Supplemental Information 3 — The data set is divided into infrared images and visible images, each type of image has 42, and one-to-one correspondence. [file peerj-cs-10-2569-s003.zip › TNO dataset/vi/33.png]

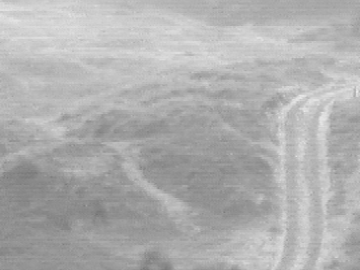

Supplement: Supplemental Information 3 — The data set is divided into infrared images and visible images, each type of image has 42, and one-to-one correspondence. [file peerj-cs-10-2569-s003.zip › TNO dataset/vi/34.png]

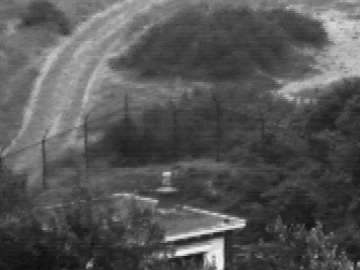

Supplement: Supplemental Information 3 — The data set is divided into infrared images and visible images, each type of image has 42, and one-to-one correspondence. [file peerj-cs-10-2569-s003.zip › TNO dataset/vi/35.png]

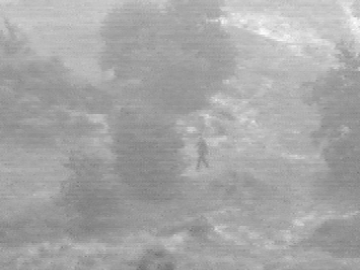

Supplement: Supplemental Information 3 — The data set is divided into infrared images and visible images, each type of image has 42, and one-to-one correspondence. [file peerj-cs-10-2569-s003.zip › TNO dataset/vi/36.png]

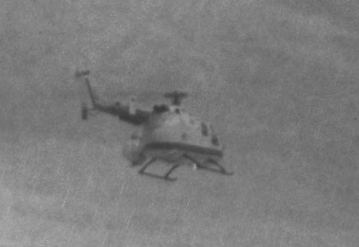

Supplement: Supplemental Information 3 — The data set is divided into infrared images and visible images, each type of image has 42, and one-to-one correspondence. [file peerj-cs-10-2569-s003.zip › TNO dataset/vi/37.png]

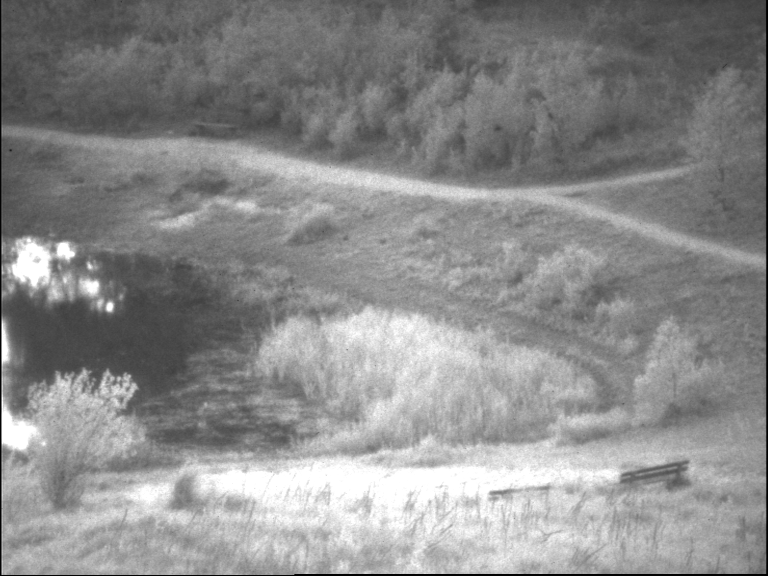

Supplement: Supplemental Information 3 — The data set is divided into infrared images and visible images, each type of image has 42, and one-to-one correspondence. [file peerj-cs-10-2569-s003.zip › TNO dataset/vi/38.png]

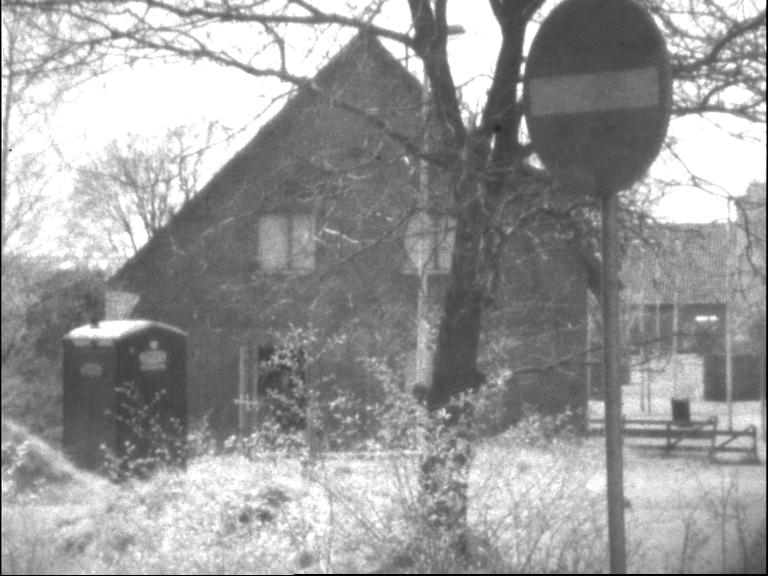

Supplement: Supplemental Information 3 — The data set is divided into infrared images and visible images, each type of image has 42, and one-to-one correspondence. [file peerj-cs-10-2569-s003.zip › TNO dataset/vi/39.png]

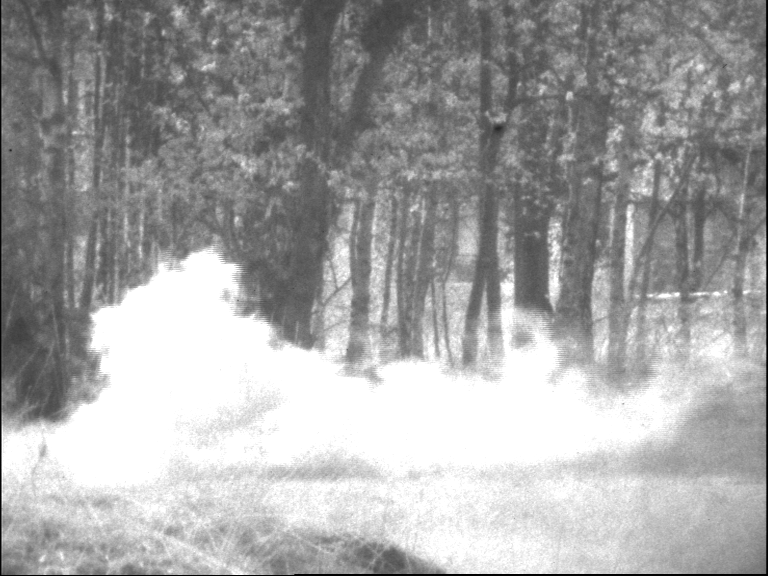

Supplement: Supplemental Information 3 — The data set is divided into infrared images and visible images, each type of image has 42, and one-to-one correspondence. [file peerj-cs-10-2569-s003.zip › TNO dataset/vi/40.png]

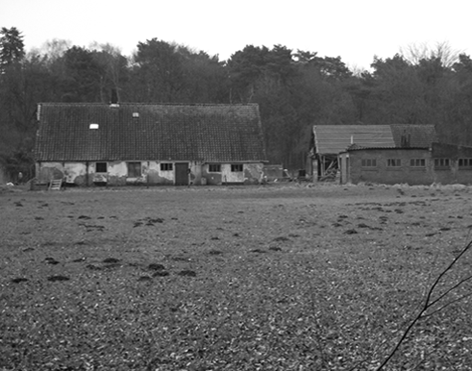

Supplement: Supplemental Information 3 — The data set is divided into infrared images and visible images, each type of image has 42, and one-to-one correspondence. [file peerj-cs-10-2569-s003.zip › TNO dataset/vi/41.png]

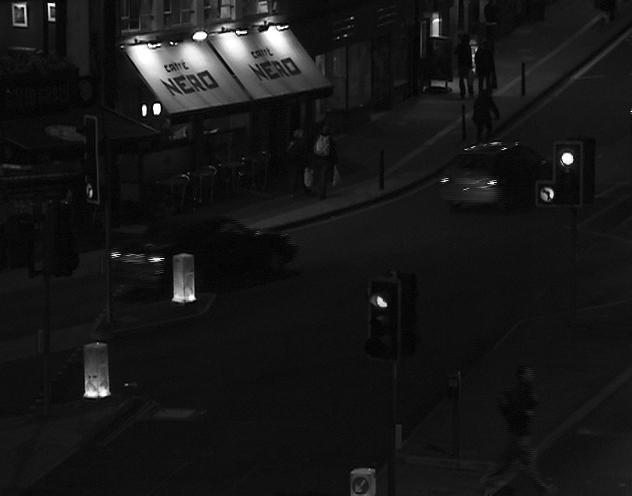

Supplement: Supplemental Information 3 — The data set is divided into infrared images and visible images, each type of image has 42, and one-to-one correspondence. [file peerj-cs-10-2569-s003.zip › TNO dataset/vi/42.png]
